# Maintaining Vital Medical Education Programs Despite the Devastating Effects of the Sudanese War

**DOI:** 10.4269/ajtmh.25-0004

**Published:** 2025-05-20

**Authors:** Mamoun Homeida, Suzan Homeida, Arwa Abubaker, Hassan Mukhtar, Charles D. Mackenzie

**Affiliations:** ^1^ University of Medical Sciences and Technology, Khartoum, Sudan;; ^2^ The END Fund, New York, New York;; ^3^ The Task Force for Global Health (TFGH), Atlanta, Georgia

## Abstract

The ongoing civil war in Sudan has had disastrous effects on all aspects of life for the Sudanese, including the essential training of new medical personnel, who are an integral component of health systems in a country in great need of healthcare. The efforts of a leading Khartoum medical university, the University of Medical Sciences and Technology, to sustain the training of medical and allied health personnel after the April civil war in 2023 serve as a unique example of adaptation and inter-country collaboration. Efforts to maintain the educational programs for well over 3,000 medical students, individuals who are desperately needed to provide healthcare in their war-torn country, are described in this report.

The continuing and often escalating wars and civil conflicts across the world result in the massive displacement of people, leading to significant human, social, and health concerns that can persist for many years. In 2024, it was estimated that 122.6 million people were displaced, with 43.7 million classified as refugees, primarily from specific stressed countries or regions.^1^ The lives of younger generations, vital to their countries’ futures, are now in turmoil, disrupting their essential education and professional development. Although many international agencies help to address the educational needs of the displaced, university-level training suffers the most. For example, the training of medical personnel, a profession much needed in countries affected by war, is usually severely, if not permanently, interrupted. This training requires physical facilities, including laboratories and hospitals, as well as trained teachers, which are generally not available in refugee camps and often not accessible in the countries to which refugees migrate.

Higher education has always been strong in Sudan, with the first school, Gordon Memorial College, established in 1908, and the Kitchener School of Medicine, established in 1924.^2^ Before the war in April 2023, there were ∼52 public and private universities in Khartoum State, most of which offered a medical degree.^3^ The war caused everyday life in Sudan to come to a sudden halt, resulting in a massive exodus of people to neighboring countries and beyond. Al Mashreg University was bombed and destroyed. Other university campuses were converted into military bases by the fighting factions, their premises looted, and essential equipment destroyed or stolen and taken out of the country, leaving virtually no chance for a quick recovery of medical education, even if the war were to end.^4^^,^^5^

The University of Medical Sciences and Technology (UMST), an institution with 14 colleges and 23 postgraduate courses, was established in 1996. When the recent war broke out, the education of 3,930 undergraduate and 385 postgraduate students attending UMST was jeopardized. Many medical, dental, and pharmacy students were near the final steps in their studies—final examinations and graduation—and their hopes of contributing to the healthy future of their country were seemingly shattered. However, UMST has always taken responsibility for its medical and allied science students. In previous times of national revolution and rioting, the university took great care to ensure the continuing education of its students. During the coronavirus disease 2019 pandemic, UMST learned how to use online teaching to address at least some of the critical needs in education services previously met by the physical presence of teachers and students. However, online and simulation-based teaching do not replace the unique educational requirements of medical training—actual patients, the dissection of cadavers, and hospital fieldwork; these are aspects of particular importance to medical students in developing countries. In Khartoum, as the war progressed, there was an ever-dwindling number of the original UMST academic staff available to continue teaching and no internet service for online instruction.

The solution to this urgent educational challenge for UMST students was to seek potential reference hospitals and universities in neighboring countries where students could be trained according to the established curriculum of UMST; thereby ensuring that students obtain their university degree, a qualification that is recognized and accredited by many international professional bodies. The University of Medical Sciences and Technology leadership requested assistance from four Arab and three African countries to accommodate the students’ needs (e.g., training hospitals, laboratories, and dissecting rooms). Recognizing that most universities have limited student placements available, various solutions were suggested, including weekend and afternoon lectures and practical sessions, as well as the donation of dental chairs and other equipment by UMST. It was also proposed that the teaching and supervision of Sudanese students could be supported by academic staff from UMST, thus addressing any shortages in the availability of lecturers at the host universities. However, Arab countries required complicated and expensive procedures for any transfer, which would likely be too costly for both students and the UMST administration. In contrast, Rwanda and Tanzania welcomed the hosting of students; university and hospital administrations, as well as government officials, were friendly and collaborative, even reaching the Presidential level in Rwanda.

Transferring students from one university to another is complicated and governed by local and international regulations, necessitating the assurance of degree equivalence and matching curricula. In this case, there was an urgent need to safeguard the academic careers of Sudanese students, which required making arrangements as quickly as possible to protect their educational and professional progress. Tanzanian hospitals were welcoming and encouraging during the negotiations, and a similar situation occurred in Rwanda, where the government, along with the University of Rwanda and its hospitals came to the aid of UMST by covering the entire cost of training.

The reception of the first batch of students in Tanzania was a significant occasion, not only as an academic event but also as a major political occasion. The Tanzanian Government proudly viewed the event as a significant example of an African country assisting a sister country in need, rescuing an essential group of refugees—medical students—and showing African solidarity. A major local newspaper (the Daily News) and social media featured the arrival of the Sudanese students. Currently, 425 students are studying at the two branches of Muhimbili Teaching Hospital (Ministry of Health) and two regional hospitals, Shree Hindu Mandal and Temeke hospitals, where they attend clinics and bedside teaching, along with regular monthly assessment examinations. A total of 184 preclinical students also receive education in anatomy, biochemistry, and physiology at Muhimbili University of Health and Allied Sciences (MUHAS), provided by both UMST and MUHAS staff. Furthermore, their former Sudanese UMST staff, now in Egypt, the Gulf countries, the United Kingdom, and the USA, continue to provide online teaching on Saturdays and Sundays.

Housing the students was a concern, especially for families who moved with their children. To allow students time to select accommodation, all students were housed for 3 days in hotels at the university’s expense and given $400 to cover the costs of finding longer-term accommodation. In Tanzania, UMST assisted by renting a hostel and guaranteeing payment for those students who opted to live in groups in furnished apartments near the hospital. The local community around the university hospital welcomed the newcomers and supported them as fellow Africans in need.

In 2024, 167 clinical students in Rwanda completed their final year and graduated. During the year an old teaching institution was converted to house the needed teaching facilities for the UMST medical students (Figure 1). The University of Medical Sciences and Technology upgraded the dental services in two regional hospitals (Kabutare and Nyanza) by providing 10 new dental chairs, radiology services, and necessary consumables as a show of support for the latest collaboration. Senior dental staff and newly trained doctors from Sudan joined the workforce in these hospitals, leading to an almost tenfold increase in patient flow after the arrival of 145 new dental students. This upgrade of hospital activities and staff was extremely well received by both the local community and the hospitals themselves. A partnership was also developed with the University of Rwanda at the Huye Campus outside Kigali, where 120 preclinical students are studying.

**Figure 1.**
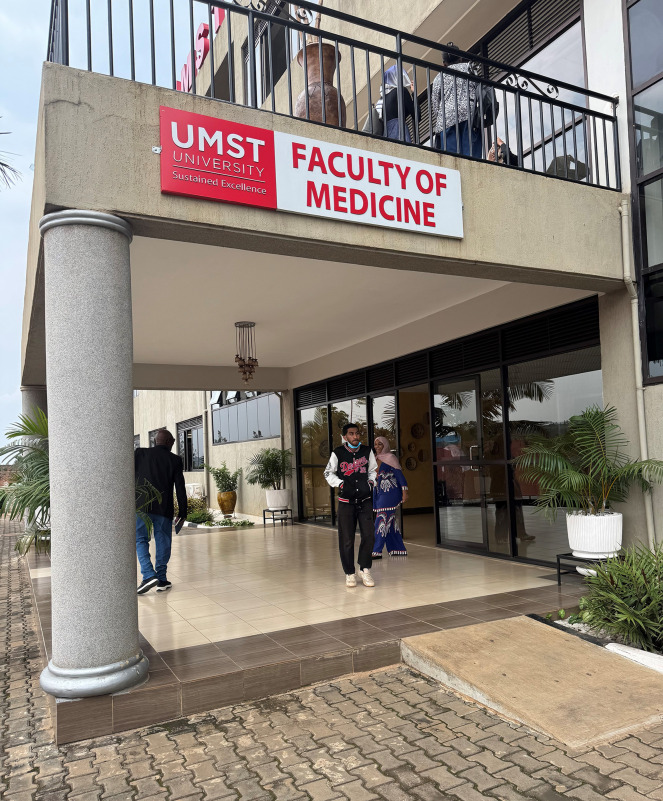
The new UMST teaching facilities in Kigali, Rwanda, established in late 2024.

Academic offices to support required academic examinations were established in the two hosting countries, as well as Egypt, Riyadh, Saudi Arabia, and Dubai, thus accommodating the diaspora of UMST students at all levels and stages of their courses, regardless of where they were located after fleeing the war. University of Medical Sciences and Technology staff who are now residents in these countries manage the arrangements for these examinations. Since the start of the war, more than 3,000 students have received the examinations they needed from 14 faculties in these centers. Final graduation examinations were given priority, although there were unique challenges with examinations requiring patients. In Rwanda, the dental school of the University of Rwanda generously donated their facilities, and 39 dental doctors have now successfully graduated.

In summary, there were many challenges in maintaining and completing the educational programs for UMST students. The success to date is due to a combination of the extraordinary dedication and commitment of the UMST staff to provide high-quality education to their students, along with the genuine support of the accommodating countries. The overall experience underscores the many challenges of relocating medical students with special instructional needs. Differences in societal norms and language can be difficult for students, and these challenges are particularly pronounced when such relocations occur at the necessary speed of fleeing an ongoing war. Nevertheless, young and focused students have a remarkable ability to adapt and learn the language; UMST provided online sessions in Swahili. It is incumbent upon appropriate agencies, such as the Organization of African Unity and the United Nations High Commissioner for Refugees, to aid these relocation activities because new medical professionals are vital to countries under civil stress. The positive inter-country activities experienced by UMST are most welcome, and the newly graduated doctors have been readily deployed across Africa, where they are able to help their new countries and support their own families. These experiences bode well for ensuring a new generation of medical professionals and their much-needed contributions toward improving health across Africa. Doctors, dentists, and pharmacists are all essential for Sudan as it recovers from the current war.
